# Relational non-local means for image denoising by cross-scale self-similarity of directional intensity-difference profiles

**DOI:** 10.1016/j.mex.2026.104230

**Published:** 2026-10-23

**Authors:** Md. Abid Hasan Rafi, Mst. Fatematuj Johora, Md. Delowar Hossain, Md. Motaharul Islam, Md. Murad Kabir Nipun, Tangina Sultana

**Affiliations:** a Department of Electronics and Communication Engineering, Hajee Moammad Danesh Science and Technology University, Dinajpur, 5200, Bangladesh; b Department of Computer Science and Engineering, Hajee Moammad Danesh Science and Technology University, Dinajpur, 5200, Bangladesh; c Department of Computer Science and Engineering, United International University, Dhaka, 1212, Bangladesh; d Department of Electrical and Electronic Engineering, International University of Business Agriculture and Technology, Dhaka, 1230, Bangladesh

**Keywords:** Image denoising, Multi-scale directional profiles, Structure-adaptive filtering, Edge preservation, Training-free denoising

## Abstract

Non-local means remove noise by averaging pixels with similar neighborhoods, but raw patches become unreliable as noise increases. This article replaces the patch with a relational descriptor: centered intensity differences sampled along K rays at S geometric radii, plus one lightly smoothed intensity value. Multi-radius sampling and arc averaging partly denoise the descriptor before matching, while centering removes noise common to all samples. A per-pixel confidence field combines cross-scale stability of the normalized profile, whose distribution under pure noise is closed-form, with a noise-corrected local contrast term to control filtering strength. The proposed method outperforms non-local means by 1.0–1.6 dB PSNR on grayscale benchmarks and ∼2 dB on color BSD68, without training and with the same asymptotic cost as non-local means, at about 2 to 4 times its measured run time in the present implementation, recovering ∼two-thirds of the NLM–BM3D gap.

• A relational matching descriptor for non-local denoising: multi-scale directional intensity-difference profiles that are contrast normalizable, partly pre-denoised, and compared per pixel with no spatial patch.
• Training-free structural confidence adaptively controls per-pixel filtering in the complexity class of NLM (about 2 to 4 times its measured run time), validated against NLM and BM3D on standard grayscale and color benchmarks.

## Graphical abstract

**Figure:**
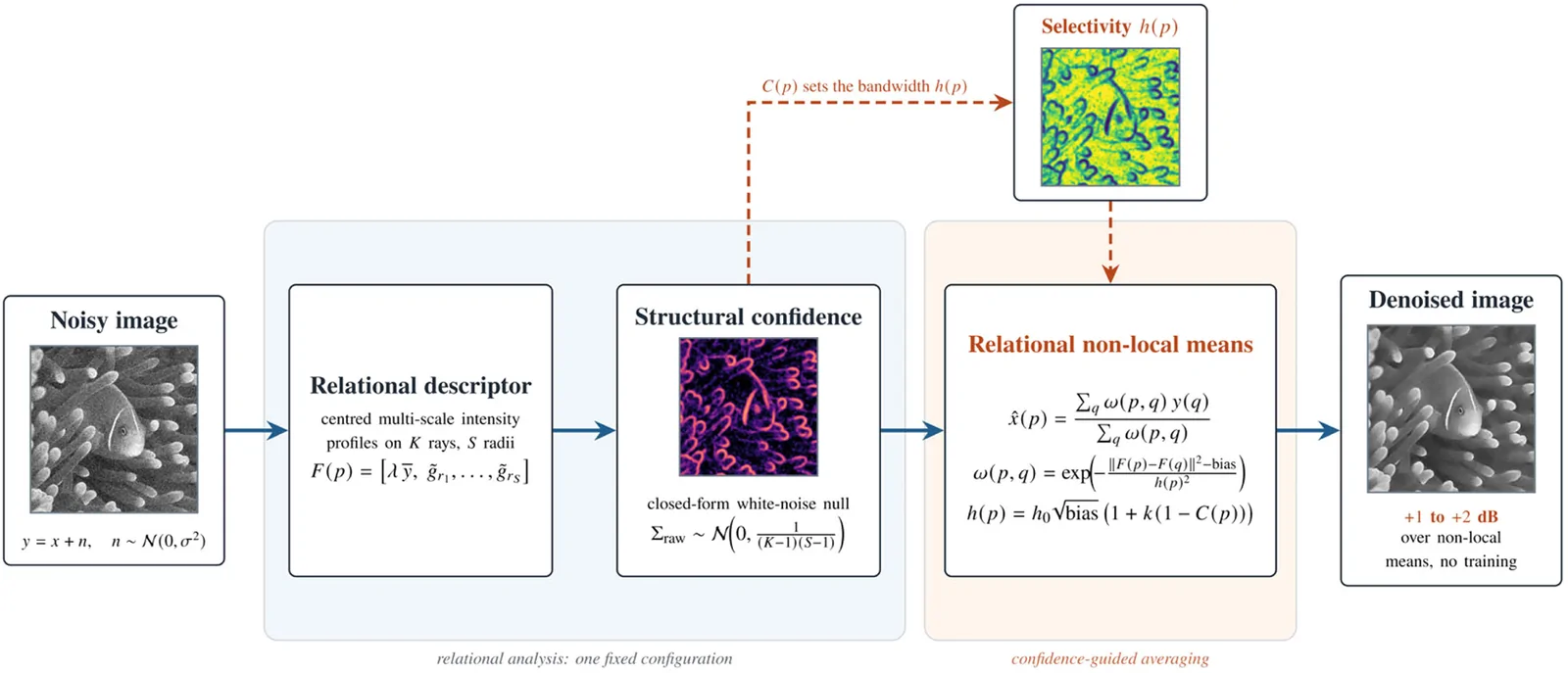

**Specifications table**

**Table:** 

Subject area	Computer Science
**More specific subject area**	Image processing and computer vision; non-local image denoising
**Name of your method**	Relational non-local means (R-NLM)
**Name and reference of original method**	Non-local means. A. Buades, B. Coll, J.-M. Morel, A non-local algorithm for image denoising, in: Proc. IEEE Conf. on Computer Vision and Pattern Recognition (CVPR), 2005, pp. 60-65.
**Resource availability**	Python reference implementation and all validation scripts: https://github.com/abidhasanrafi/crisp-denoise.

## Background

Image denoising is a standard early step in many imaging pipelines, and the choice of denoiser is often fixed by the deployment setting rather than by peak accuracy. Learned denoisers based on convolutional networks and transformers define the current state of the art [[1], [2], [3], [4]], but they need a training set of matched noisy and clean images, a graphics processor for practical throughput, and a model that transfers to the noise present at test time [5]. Block-matching and 3D filtering (BM3D) [6,7] reaches close to learned accuracy without training, at the cost of a grouped three-dimensional transform, a hard-thresholding stage, and a Wiener stage, which together make it several times slower than simpler filters. When none of that is available, practitioners use the classical linear and edge-aware filters: Gaussian smoothing, the bilateral filter, total variation, and non-local means [8]. These run in near-linear or windowed-linear time and need no training, but they remove noise by assuming local smoothness or patch self-similarity, and both assumptions weaken as the noise level rises, so structure is lost. Recent surveys describe the accuracy and cost trade-off across these families [4,9].

Non-local means is the most capable member of the training-free group. It estimates each pixel as a weighted average of pixels in a search window, with weights set by the Euclidean distance between the small patches centered on the two pixels. The limitation is the patch itself. At the signal-to-noise rastio of moderate noise the patch distance is dominated by noise variance, which forces a bias correction and a wide averaging kernel, and the fixed patch size represents oriented structure at only one scale. Learned variants fold non-local aggregation into a network and recover accuracy, but they reintroduce training [1,3].

For reference, the two baselines are as follows. Non-local means [8] estimates the value at p as a weighted average of the noisy values in a search window W(p), with weights exp(−max(d²(p,q) − 2σ², 0)/h²), where d²(p,q) is the mean squared difference between the patches centered at p and q and h is a filter parameter. A direct evaluation compares two patches of 25 values (5 by 5) for each of |W| candidates per pixel, which costs O(n·|W|·25) for n pixels. Running-sum implementations, such as the fast mode of scikit-image [10] used here, obtain the patch distance of every pixel for one offset from a box filter of the squared difference image and so cost O(n·|W|) with a small constant. BM3D [6,7] runs in two stages, hard thresholding and then Wiener filtering. For each reference block of 8 by 8 pixels, taken on a grid with a step of a few pixels, it searches a window of about 39 by 39 positions for up to 16 (first stage) or 32 (second stage) similar blocks, stacks them into a three-dimensional array, applies a separable three-dimensional transform, shrinks the coefficients, inverts the transform, and returns the block estimates to their positions with aggregation weights. Block matching alone needs about 39² × 64 ≈ 10⁵ operations per reference block, and each stage adds the cost of the transforms, so BM3D costs more per pixel than either form of non-local means.

This article asks a narrower question. Keeping the non-local means cost class and using no training, how much of the structure that non-local means discards can be recovered by changing only the descriptor used for matching and the rule that sets the local filter strength? The method below, relational non-local means, answers with two components. The first is a relational descriptor built from intensity differences sampled along rays at several radii; it is contrast normalizable, partly denoised by construction, and sensitive to oriented structure at each radius. The second is a per-pixel confidence field, derived from the cross-scale stability of that descriptor and from a noise-corrected contrast measure, with a known distribution under pure noise, that scales the filter selectivity. On three grayscale test sets and one color test set the method improves on non-local means by about 1 to 2 dB in peak signal-to-noise ratio across noise levels without changing the asymptotic cost. The measured run time of the present implementation is about 2 to 4 times that of a compiled non-local means (Section 8 of Method validation).

## Method details

Relational non-local means keeps the structure of non-local means, a weighted average of pixel values over a local search window, and changes two things. Pixels are compared through a relational descriptor rather than a raw patch, and the averaging strength is set separately for each pixel from a confidence field with a known behavior under pure noise. The three parts are the relational profiles (Section 1), the confidence field (Section 2), and the filter (Section 3). Every parameter is fixed to one setting for all noise levels and all test sets.

### Multi-scale directional relation profiles

Let I be the noisy image on the 0 to 255 intensity scale. Fix K sampling directions uₖ = (cos θₖ, sin θₖ) with θₖ = 2πk / K for k = 0,…,K − 1, and S sampling radii r₁ < ⋯ < r_S spaced geometrically. For a pixel p and a radius r the raw profile is the length-K vector of intensity differences

(1) $$g_{r}\left(p\right)\left[k\right]=A_{r}\left(p+ru_{k}\right)-I\left(p\right),k=0,\ldots ,K-1$$

where Aᵣ(x) is the mean of three bilinearly interpolated samples spread by about one pixel along the sampling circle at x. The arc average lowers the variance of each entry without smoothing across the circle, because all three taps lie at the same radius. The three taps sit at the angles θₖ − 1/r, θₖ, and θₖ + 1/r, in radians, which puts them one pixel apart along the circle. Each tap is a bilinear interpolation of the four nearest pixels. Samples that fall outside the image are read from a copy of the image padded by ⌈r⌉ + 2 pixels with mirror reflection that does not repeat the border pixel (the reflect mode of NumPy). The Gaussian filters used elsewhere in the method (the smoothed intensity, the spatial average in Section 2.1, and the gradient-energy map in Section 2.2) and the Sobel operator use the mirror mode of SciPy, in which the border pixel is repeated.

Two operations turn gᵣ(p) into the quantity used for matching. First it is centered,

(2) $${\tilde{g}}_{r}\left(p\right)\;=\;g_{r}\left(p\right)-\frac{1}{K}\sum_{k=0}^{K-1}g_{r}\left(p\right)\left[k\right]$$

which removes the term −I(p) common to every entry. Under additive white noise this term carries the noise of the center pixel; after centering, profiles measured at different radii depend on disjoint samples and are independent. This is exact for point samples on distinct pixels and only approximate for the interpolated, arc-averaged samples used here (Section 2.1). Second, for the confidence field only, the centered profile is scaled to unit length, ${\hat{g}}_{r}\left(p\right)={\tilde{g}}_{r}\left(p\right)\;/\;∥{\tilde{g}}_{r}\left(p\right)∥$, which drops local contrast and keeps the shape of the difference pattern. Sampling at S radii and averaging along each arc means the descriptor is already partly denoised before any comparison. A single S by K profile also encodes oriented structure at several distances from the pixel, which a fixed small patch cannot.

### Structural confidence field

#### Cross-scale stability

The first component measures whether the shape of the normalized profile is stable as the sampling radius changes. Real edges, ramps, and periodic textures produce a shape that changes slowly with radius; noise produces an independent shape at each radius. The statistic is the mean cosine similarity of consecutive normalized profiles,

(3) $$\Sigma_{\text{raw}}\left(p\right)\;=\;\frac{1}{S-1}\sum_{s=1}^{S-1}\langle \;{\hat{g}}_{r_{s}}\left(p\right),\;{\hat{g}}_{r_{s+1}}\left(p\right)\;\rangle$$

followed by a Gaussian spatial average of standard deviation ρ, $\Sigma \left(p\right)=\left(G_{\rho }*\Sigma_{\text{raw}}\right)\left(p\right)$. Under the null model of a flat region corrupted by independent Gaussian noise, and after centering by (2), ${\hat{g}}_{r_{s}}\left(p\right)$ and ${\hat{g}}_{r_{s+1}}\left(p\right)$ are independent isotropic unit vectors in the (K − 1)-dimensional subspace orthogonal to the constant vector, to which centering restricts them. The inner product of two such vectors has mean zero and variance 1/(K−1). Averaging S−1 cosine terms, which are mutually independent under the null (see below), gives

(4) $$\Sigma_{\text{raw}}\;∼\;N\left(0,\;\frac{1}{\left(K-1\right)\left(S-1\right)}\right)$$

For K = 8 and S = 3 the predicted standard deviation is 0.267, and for K = 16 and S = 3 it is 0.183. Monte Carlo runs on pure noise with the implemented sampler give mean 0.013 and standard deviation 0.282 for K = 8 with three arc taps, and mean 0.022 and standard deviation 0.194 for K = 16 with one arc tap (Section 2 of Method validation). The spatial average narrows the distribution further, but less than independence would give: neighboring values of Σ_raw share samples, and for ρ = 2.5 the measured standard deviation after averaging is 0.062, against 0.021 if the roughly 4πρ² = 79 pixels under the kernel were independent. A closed-form null lets Σ be thresholded at a chosen false-positive rate without any calibration data.

**Assumptions behind (4).** The closed form is exact under three assumptions: the noise is white and Gaussian, the samples entering the two profiles are values of distinct pixels, and the profile is centered. The implemented sampler departs from the second assumption. Bilinear interpolation and the three-tap arc average make each sample a weighted sum of several pixels, so neighboring entries of one profile, and entries at the two smallest radii, are positively correlated. The distribution of Σ_raw is then not exactly the stated one: the measured standard deviation is about 5 percent above the closed form in both settings (0.282 against 0.267, and 0.194 against 0.183), and the mean is shifted upward by 0.01 to 0.02. Consecutive cosine terms share one normalized profile, but this does not correlate them. For independent isotropic profiles the distribution of ⟨ĝₛ, ĝₛ₊₁⟩ given ĝₛ does not depend on ĝₛ, so the terms are independent, and the measured correlation between the two terms at K = 8 is −0.001. Thresholds should therefore be taken from the measured null, or from (4) with a margin of about 5 percent. In the filter, C enters as a continuous weight, so the null serves for calibration and interpretation and not as a hard test.

#### Noise-corrected contrast

Cross-scale stability responds weakly to a sharp edge in light noise, where a plain gradient already separates edge from background. The second component supplies that signal. Let $E\left(p\right)=\left(G_{2}*∥\nabla I{∥}^{2}\right)\left(p\right)$ be a smooth gradient-energy map. Because noise raises E everywhere by an approximately constant amount, a floor $\varphi (\hat{\sigma })$ is subtracted before E is used. The floor is the mean of E computed by passing a synthetic Gaussian noise field of the estimated standard deviation $\hat{\sigma }$ through the same operator. The contrast term is

(5) $$\text{con}\left(p\right)=\frac{m\left(p\right)}{m\left(p\right)+q_{90}},m\left(p\right)=\text{max}\left(E\left(p\right)-\varphi \left(\hat{\sigma }\right),0\right)$$

where q₉₀ is the 90th percentile of m over the image. In a flat noisy region con(p) is near zero; at an edge it approaches one. Because con(p) = 0.5 where m(p) equals its 90th percentile, the squashing scale follows each image and does not depend on contrast or noise level.

#### Fusion

The gradient term is reliable when noise is low and the stability term is reliable when noise is high, so the two are combined with a noise-dependent weight,

(6) $$w\left(\hat{\sigma }\right)\;=\;\text{clip}\left(\frac{\hat{\sigma }-8}{44},\;0.15,\;0.85\right)$$

(7) $$C\left(p\right)\;=\;\text{clip}\left(\;w\left(\hat{\sigma }\right)\;\text{clip}\left(\Sigma \left(p\right),0,1\right)\;+\;\left(1-w\left(\hat{\sigma }\right)\right)\;\text{con}\left(p\right),\;0,\;1\right)$$

The noise standard deviation σ̂ is estimated from the median absolute deviation of wavelet detail coefficients as implemented in scikit-image [10], with a direct median-absolute-deviation fallback. C(p) is near one on structure and near zero on noise. Fig. 1 shows C and the map it produces.

**Fig. 1:**
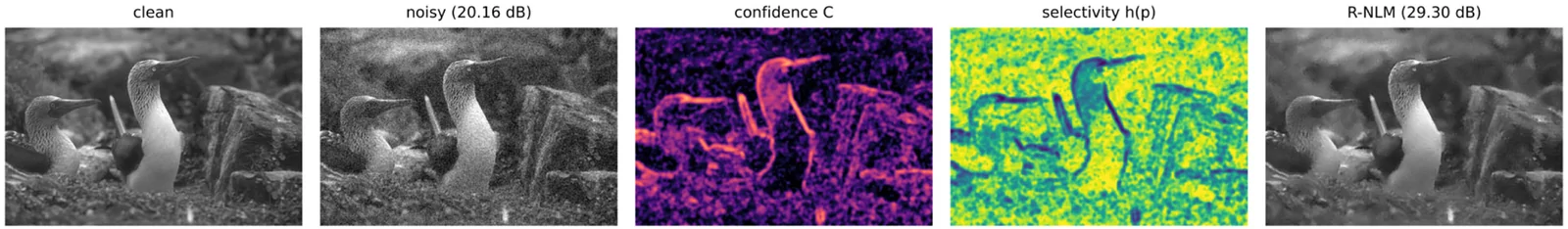
Explainability, σ = 25. From left: clean image, noisy input, confidence field C, per-pixel selectivity h(p), and the R-NLM output. C is high on contours and texture and low on the flat background; h(p) is its inverse.

**Choice of constants.** The constants in (6) are empirical. The gradient term is the more reliable one at low noise and the stability term at high noise, so w(σ̂) rises linearly with σ̂ and is clipped at 0.15 and 0.85 so that neither term is switched off. The ramp gives w = 0.16, 0.39, and 0.85 at σ̂ = 15, 25, and 50. The slope and end points were set by hand in a preliminary comparison on 13 grayscale test images bundled with scikit-image, where the weighted sum reached an area under the ROC curve of 0.92, 0.88, and 0.81 at σ = 10, 25, and 50, against 0.80, 0.81, and 0.78 for the stability term alone. The width ρ = 2.5 of the spatial average was the largest single gain in the phantom separation study, where Cohen's d rose from 2.3 to 7.7 at σ = 25. In the filter these values matter little: replacing C by the stability term alone changed the PSNR by less than 0.05 dB on the eight ablation images. Table 1 lists this and every other constant with the basis for its value.

**Table 1:** Parameters of the method, their values, and the basis for each value.

Parameter	Value	Role	Basis for the value
K	8	Number of ray directions per profile	K = 16 lowered the gain by about 0.2 dB (Table 6).
S; radii rₛ	3; 1.5, 3.0, 6.0 pixels	Distances at which differences are sampled	Geometric ratio of 2, smallest radius above one pixel, largest below R = 7. Two radii changed the gain by +0.1 to −0.2 dB; the third radius helps only at σ = 50 (Table 6).
Arc taps	3	Samples averaged along each arc, one pixel apart	Lowers the variance of each entry; five taps stayed within 0.2 dB (Table 6).
ρ	2.5 pixels	Width of the spatial average of Σ_raw	Largest gain in the phantom separation study (Cohen's d from 2.3 to 7.7 at σ = 25).
Gradient-energy smoothing	2.0 pixels	Width of the Gaussian in E(p), Eq. (5)	Set once and not tuned.
Percentile in Eq. (5)	90	Scale of con(p)	Makes con = 0.5 where m equals its 90th percentile; not tuned.
w(σ̂), Eq. (6)	ramp (σ̂ − 8)/44, limits 0.15 and 0.85	Balance of the stability and contrast terms	Set by hand from the behavior of the two terms at low and high noise; AUC 0.92, 0.88, 0.81 against 0.80, 0.81, 0.78 for stability alone (13 images). Using stability alone changed PSNR by less than 0.05 dB.
Intensity smoothing	0.7 pixel	Gaussian width of the intensity entry	Carried over from earlier experiments with the confidence gate; not tuned for the filter.
λ	2.2	Weight of the intensity entry	Peak of a plateau at 2.2 to 2.5. λ = 1.0 lowered the gain by 0.7 to 1.1 dB; 1.6 to 3.0 stayed within 0.2 dB (Table 6).
h₀	0.6	Base selectivity, in units of √bias	h₀ = 0.45 lowered the gain by 0.4 to 0.5 dB; 0.75 stayed within 0.2 dB (Table 6).
k	0.8	Extra smoothing where C is low, Eq. (9)	k = 0 lowered the gain by 0.8 to 1.1 dB; 0.4 to 1.5 stayed within 0.2 dB (Table 6).
R	7	Half-width of the search window	Equals the 15 by 15 window of the non-local means baseline; R = 5 stayed within 0.2 dB.

Ablation values refer to Table 6 (BSD68, 8 images, σ = 15, 25, 50). Those eight images are also the parameter selection images.

### Relational non-local means filter

#### Feature vector

For matching, each pixel is described by

(8) $$F\left(p\right)\;=\;\left[\;\lambda \;z_{0}\left(p\right),\;\;z_{1}\left(p\right),\;\ldots ,\;z_{S}\left(p\right)\;\right]\;\in \;R^{1+KS}$$

the standardized smoothed intensity z₀, scaled by an anchor weight λ, followed by the standardized centered profiles zₛ of the S radii. Here z₀(p) = G₀.₇(I)(p) / (s₀σ̂) and zₛ(p)[k] = g̃ᵣₛ(p)[k] / (sₛ,ₖσ̂), G₀.₇ is a Gaussian filter of standard deviation 0.7 pixel, and s₀ and sₛ,ₖ are the standard deviations of the same quantities computed once on a 160 by 160 field of independent standard normal noise with a fixed seed. The intensity entry is required, because the centered profiles are blind to absolute level and without it pixels of similar texture but different brightness would be averaged together. An ablation with the profiles removed, that is matching on the smoothed intensity alone, loses 0.3 to 2.7 dB (Section 7 of Method validation). Dividing by sσ̂ gives every entry unit variance under white noise of the estimated level. The anchor weight λ is applied after this standardization, so it changes the weight of the intensity entry in the distance and leaves the standardization unchanged.

**Distance offset.** Write F(p) = μ(p) + ε(p), the noise-free feature plus the noise part. By construction every entry of ε has variance one, except the intensity entry, whose variance is λ². For two pixels with the same noise-free feature, ‖F(p) − F(q)‖² = ‖ε(p) − ε(q)‖², and its expectation is E‖ε(p)‖² + E‖ε(q)‖² − 2E⟨ε(p), ε(q)⟩. The first two terms add to 2(KS + λ²) whatever the correlation between different entries of one descriptor, because only the variance of each entry enters; the KS profile entries contribute one each and the intensity entry contributes λ². This is the value bias = 2(KS + λ²). The third term vanishes when the noise in the two descriptors is uncorrelated, which holds for candidates whose sampling supports do not overlap and only approximately for close neighbors. On a white-noise field with σ = 25, the mean of ‖F(p) − F(q)‖² divided by bias was 0.57 for a horizontal offset of one pixel, 0.80 for a diagonal offset of one pixel, 0.97 for two pixels, and between 1.00 and 1.04 for offsets of three pixels or more. For the nearest neighbors the subtracted offset therefore exceeds the noise contribution and their weights are raised, while for all other candidates it is correct to within about 4 percent. The maximum with zero in (10) handles the random fluctuation of the distance around its mean.

#### Confidence-modulated selectivity

The filter strength at pixel p is set from the confidence field,

(9) $$h\left(p\right)\;=\;h_{0}\;\sqrt{\text{bias}}\;\left(1+k\;\left(1-C\left(p\right)\right)\right)$$

so, a pixel on structure (C near one) is filtered with the base strength h₀·√(bias), and a pixel in noise (C near zero) is filtered up to 1 + k times more strongly.

#### Weighted average

The output is a weighted average of the noisy values over a square window W of half-width R,

(10) $$\omega \left(p,q\right)\;=\;\text{exp}\left(-\;\frac{\text{max}\left(∥F\left(p\right)-F\left(q\right){∥}^{2}-\text{bias},\;0\right)}{h{\left(p\right)}^{2}}\right)$$

(11) $$\hat{J}\left(p\right)\;=\;\frac{\sum_{q\;\in \;W\left(p\right)}\omega \left(p,q\right)\;I\left(q\right)}{\sum_{q\;\in \;W\left(p\right)}\omega \left(p,q\right)}$$

Subtracting bias in (10) removes the noise contribution to the distance, so a perfect match receives weight one and the exponent depends only on the difference in underlying structure. The comparison in (10) is between single feature vectors, with no averaging over a spatial patch. Classical non-local means compares patches, which needs a second smoothing pass over the patch window. The relational descriptor already pools a neighborhood through the S radii, so the extra pass is not needed, and no averaging over a patch window is required. The descriptor has 1 + KS = 25 entries, however, so each candidate comparison costs more arithmetic than in a running-sum implementation of non-local means (Section 5).

In (10), h(p)² sets how quickly the weight falls as the distance exceeds the offset, and the maximum with zero gives weight one to every candidate whose distance is no larger than noise alone would produce. The candidate q = p is always in W(p) with weight one, so the denominator in (11) is at least one. Near the image border W(p) is cut to the pixels inside the image, so fewer candidates are averaged and no padded values enter the sum. The feature vectors of border pixels are computed from the mirrored samples described in Section 1. The plain-array fallback in the reference code wraps the window around the border instead; all reported results use the compiled path.

## Color images

For a three-channel image the feature vector holds the smoothed intensity of each channel, R, G, and B, followed by the centered profiles computed on the luminance channel. Let Y = 0.2989 R + 0.5870 G + 0.1140 B be the luminance. The feature is F(p) = [λz_R(p), λz_G(p), λz_B(p), z₁(p), …, z_S(p)] ∈ ℝ^(3+KS), where z_R, z_G, and z_B are the standardized smoothed channels and zₛ are the standardized centered profiles of Y, so the offset becomes bias = 2(KS + 3λ²). The standardization constants are measured on a three-channel field of independent standard normal noise, so the profiles of Y are scaled by the noise that Y actually receives, and σ̂ in the standardization is the mean of the three per-channel estimates. The confidence field, including the weight w in (6), is computed from Y with the noise estimate of the luminance image. One set of weights from (10) is applied to all three channels in (11).

## Parameters and computational cost

Every result in this article uses K = 8, S = 3 with r = (1.5, 3.0, 6.0) pixels, three arc taps, ρ = 2.5, λ = 2.2, h₀ = 0.6, k = 0.8, and R = 7. This setting was chosen on the first eight BSD68 images in sorted file order (0000 to 0007), with a confirmation run on the first ten, at σ = 15, 25, and 50 and with noise realizations that differ from those of the reported evaluation. It was then frozen and used for all noise levels and all test sets. The overlap between these images and the BSD68 evaluation is discussed in Section 1 of Method validation. A sweep of each parameter (Section 7 of Method validation) shows that only λ, k, and a smaller h₀ (0.45) change the result by more than 0.2 dB, and K = 16 lowers it by about 0.2 dB, and that raising λ helps only when h₀ and k are left at their defaults. Table 1 gives the role of each constant and the basis for its value.

The feature build costs O(nKS) for n pixels. The weighted average costs O(n·|W|·(1 + KS)) with |W| = (2R + 1)² = 225 and a descriptor of 1 + KS = 25 numbers, which matches the cost of non-local means with a 5 by 5 patch. The inner loop is provided in compiled form with a plain array fallback. For comparison, direct non-local means with a 5 by 5 patch costs O(n·|W|·25), the same order and constant as the weighted average here, and the running-sum form used as the baseline costs O(n·|W|) with a small constant, because the patch distance for one offset comes from a box filter of the squared difference image. The distance in (10) has no such shortcut, since every candidate pair needs all 25 entries. Counting multiply-adds per pixel, the weighted average here needs about 5.6 × 10³ (225 × 25), against about 1 × 10⁴ for the block matching of one BM3D stage with the default block size, window, and step (39² × 64 / 3²). BM3D has two stages and the transforms in addition, so it needs at least about four times as much arithmetic. These are order-of-magnitude counts and not measurements; the measured times are in Section 8 of Method validation.

## Reference implementation

A Python implementation using NumPy, SciPy, and an optional compiled inner loop is available at: https://github.com/abidhasanrafi/crisp-denoise. The package exposes a single function that denoises a grayscale or color array, and the validation scripts reproduce every table and figure in this article from fixed random seeds. Algorithm 1 gives the full single pass procedure of relational non-local means.

**Algorithm 1:** Relational non-local means (single pass).

**Input:** noisy image I; K ray directions; radii r₁ < … < rₛ; aggregation width ρ; anchor weight λ; base exponent h₀; modulation k; window radius R. Constants s₀ and sₛ: unit-noise standard deviations of the feature columns (Section 3.1).
**Output:** denoised image Ĵ.
1: σ̂ ← estimateNoise(I) // median absolute deviation
2: **for** each radius r ∈ {r₁,…,rₛ} do
3: s_r ← arcAverage(I, r); g̃_r ← subtractMean(s_r − I) // Eq. (1)-(2)
**end for**
4: Σ ← Gρ * crossScaleCosine(ĝ at r₁,…,rₛ); con ← noiseFlooredContrast(I, σ̂) // Eq. (3)-(5)
5: C ← w(σ̂)·clip(Σ) + (1 − w(σ̂))·con // Eq. (6)-(7)
6: z₀ ← G₀.₇(I) / (s₀σ̂); zₛ ← g̃ᵣₛ / (sₛσ̂), s = 1,…,S; F ← [λz₀, z₁, …, z_S] // standardize, then apply λ; Eq. (8)
7: bias ← 2(KS + λ²); h ← h₀·√bias·(1 + k(1 − C)) // Eqs. (9)-(10); color: bias ← 2(KS + 3λ²)
8: num ← 0; den ← 0
9: **for** each offset δ = (dy, dx) with |dy|, |dx| ≤ R do
10: d² ← Σ_c (F_c(p) − F_c(p + δ))² // all pixels p at once; sum over the columns c
11: ω ← exp(−max(d² − bias, 0) / h²) // Eq. (10)
12: num ← num + ω·I(p + δ); den ← den + ω
**end for**
13: Ĵ ← num / den // Eq. (11)

## Method validation

### Experimental setup

Clean images were corrupted with additive white Gaussian noise at standard deviations σ = 15, 25, and 50 on the 0 to 255 scale, and at σ = 10, 25, and 50 for the separation study. Random seeds were fixed per experiment. Two baselines were used: non-local means as implemented in scikit-image [10] with filter parameter h = 1.05·σ̂, a 5 by 5 patch and a 15 by 15 search window in fast mode, and BM3D [6,7] with the noise standard deviation supplied. Quality was measured by peak signal-to-noise ratio (PSNR), the structural similarity index (SSIM) [11], and an edge-preservation index defined as the Pearson correlation between the Laplacian responses of the clean and the restored image. Grayscale tests use the 68-image Berkeley subset (BSD68) drawn from the Berkeley Segmentation Dataset [12], the 12-image Set12, and the 24-image Kodak set. Color tests use the color version of the 68-image Berkeley subset (CBSD68). All test images are publicly available. Software: Python 3.14.3, NumPy 2.4.2, SciPy 1.17.0, scikit-image 0.26.0, Numba 0.66.0, and the bm3d package 4.0.3 [7], run on Windows 11 with a laptop CPU using four threads. R-NLM estimates the noise level as described in Section 2.3; only the parameter selection runs and the ablation in Table 6 supplied the true σ. Image subsets and seeds: the 20-, 15-, and 8-image experiments use the first 20, 15, and 8 images of BSD68 (or CBSD68 for color) in sorted file-name order (0000 to 0019, 0000 to 0014, and 0000 to 0007), and the noise seeds are fixed per experiment (100 + i for the separation study, 600 + i for the BSD68 comparison, 900 + i for the ablation, and 1000 + i for edge preservation, with i the image index). Overlap with parameter selection: the parameters were chosen on BSD68 images 0000 to 0007 (confirmed on 0000 to 0009) with other noise seeds (400 + i and 500 + i), so these images belong to the BSD68 test set and to each BSD68 subset above, and the ablation in Table 6 uses the same eight images. That ablation therefore measures sensitivity around the chosen setting and not out-of-sample accuracy. Two results address the overlap. Set12 and Kodak24 played no part in choosing the parameters, and the gains on them (Table 3) are of the same size as on BSD68. In addition, re-scoring BSD68 with the protocol of Table 3 on the 58 images that were not used for parameter selection (0010 to 0067) gives a gain over non-local means of +1.53, +1.51, and +1.08 dB at σ = 15, 25, and 50, against +1.49, +1.47, and +1.02 dB on all 68 images, and the method recovers 63, 71, and 62 percent of the gap to BM3D.

### Noise distribution of the stability statistic

A field of pure Gaussian noise was passed through Eqs. (1) to (3) with the spatial average disabled. The field was 320 by 320 with σ = 25, and K = 16, S = 3, and one arc tap were used. The stability statistic Σ_raw had empirical mean 0.022 and standard deviation 0.194, against the predicted mean of zero and standard deviation 1/√((K−1)(S−1)) = 0.183 from (4). The small positive bias comes from bilinear interpolation in the arc sampling. A phase-based statistic tested as an alternative matched a Rayleigh tail, with a false-positive rate of 0.057 at threshold 0.95 against a predicted 0.067, but it failed to separate texture from noise and was not used (Section 9). With the K = 8, three-tap sampler used for filtering, a 400 by 400 field gave mean 0.013 and standard deviation 0.282 against a predicted 0.267, the two cosine terms had a correlation of −0.001, and the standard deviation after the spatial average with ρ = 2.5 was 0.062.

### Structure versus noise separation

On BSD68 the clean image was labeled by structure-tensor energy, with a structure class (top 40 percent) and a flat class (bottom 30 percent), and the confidence field was evaluated on the noisy image. Table 2 reports the area under the receiver operating characteristic curve for the fused field C, for the stability term alone, and for a noise-corrected gradient used as a control. The fused field separates the two classes above the gradient control at every noise level, and the margin grows with noise. The stability term alone reaches only about 0.85; the two terms are complementary. On a synthetic phantom with high-contrast structure the separation is near perfect at every noise level (area above 0.99 up to σ = 50).

**Table 2:** Area under the ROC curve for structure versus flat pixels, BSD68 grayscale, 20 images.

Noise σ	Fused C (AUC)	Fused C (Cohen's d)	Stability term (AUC)	Gradient control (AUC)
10	**0.982**	2.68	0.808	0.978
25	**0.950**	2.25	0.868	0.928
50	**0.875**	1.49	0.841	0.785

BSD68, 20 images. Structure and flat pixel classes are the top 40 % and bottom 30 % of structure-tensor energy of the clean image. AUC is the area under the ROC curve; the gradient control is a noise-floor-corrected gradient magnitude. On a synthetic high-contrast phantom the fused field reaches AUC above 0.99 at every noise level.

### Grayscale denoising

The analysis in Table 3 reports PSNR and SSIM on BSD68, Set12, and Kodak. Relational non-local means improves on non-local means by 1.49, 1.47, and 1.02 dB on BSD68 at σ = 15, 25, and 50, with SSIM gains of 0.07, 0.07, and 0.05. The improvement is 0.98 to 1.42 dB on Set12 and 1.23 to 1.64 dB on Kodak. Across the three sets the method recovers about two thirds of the PSNR gap between non-local means and BM3D. BM3D stays ahead by 0.7 to 1.4 dB. BM3D applies a second, Wiener-type filtering stage that the present method does not, and a two-pass version of the present filter did not close the gap (Section 9). Fig. 2, Fig. 3.

**Table 3:** Grayscale denoising, PSNR (dB) / SSIM. R-NLM is the proposed method; BM3D is listed for reference.

Dataset	Method	σ = 15		σ = 25		σ = 50	
		PSNR	SSIM	PSNR	SSIM	PSNR	SSIM
BSD68 (68 images)	Noisy	24.61	.580	20.17	.406	14.15	.205
	NLM [8]	28.73	.784	26.47	.702	23.89	.579
	R-NLM	**30.22**	**.850**	**27.94**	**.775**	**24.91**	**.629**
	BM3D [6,7]	31.13	.880	28.61	.809	25.68	.689
	Δ (R-NLM − NLM)	+1.49	+.066	+1.47	+.073	+1.02	+.050
Set12 (12 images)	Noisy	24.62	.560	20.18	.390	14.16	.202
	NLM [8]	30.19	.839	27.44	.774	23.93	.651
	R-NLM	**31.17**	**.875**	**28.86**	**.816**	**25.34**	**.681**
	BM3D [6,7]	32.39	.900	29.98	.854	26.75	.765
	Δ (R-NLM − NLM)	+0.98	+.036	+1.42	+.042	+1.41	+.030
Kodak24 (24 images)	Noisy	24.61	.517	20.18	.341	14.16	.161
	NLM [8]	29.88	.790	27.45	.715	24.65	.599
	R-NLM	**31.41**	**.850**	**29.09**	**.777**	**25.88**	**.635**
	BM3D [6,7]	32.36	.880	29.96	.815	27.02	.709
	Δ (R-NLM − NLM)	+1.53	+.060	+1.64	+.062	+1.23	+.036

PSNR in dB, SSIM in [0, 1]. Additive white Gaussian noise. One fixed R-NLM configuration is used for every dataset and noise level. The better training-free value (NLM vs. R-NLM) in each column is in bold; BM3D is listed for reference.

**Fig. 2:**
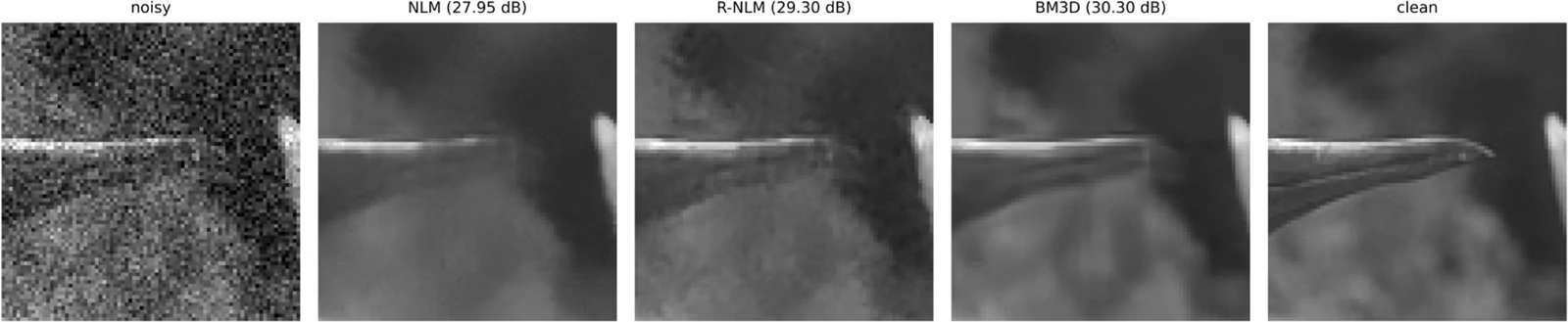
Detail crop, σ = 25. From left: noisy, non-local means, R-NLM, BM3D, clean. The R-NLM edge is sharper than non-local means and BM3D is smoother overall. A faint low-frequency patch is visible in the dark region of the R-NLM crop.

**Fig. 3:**
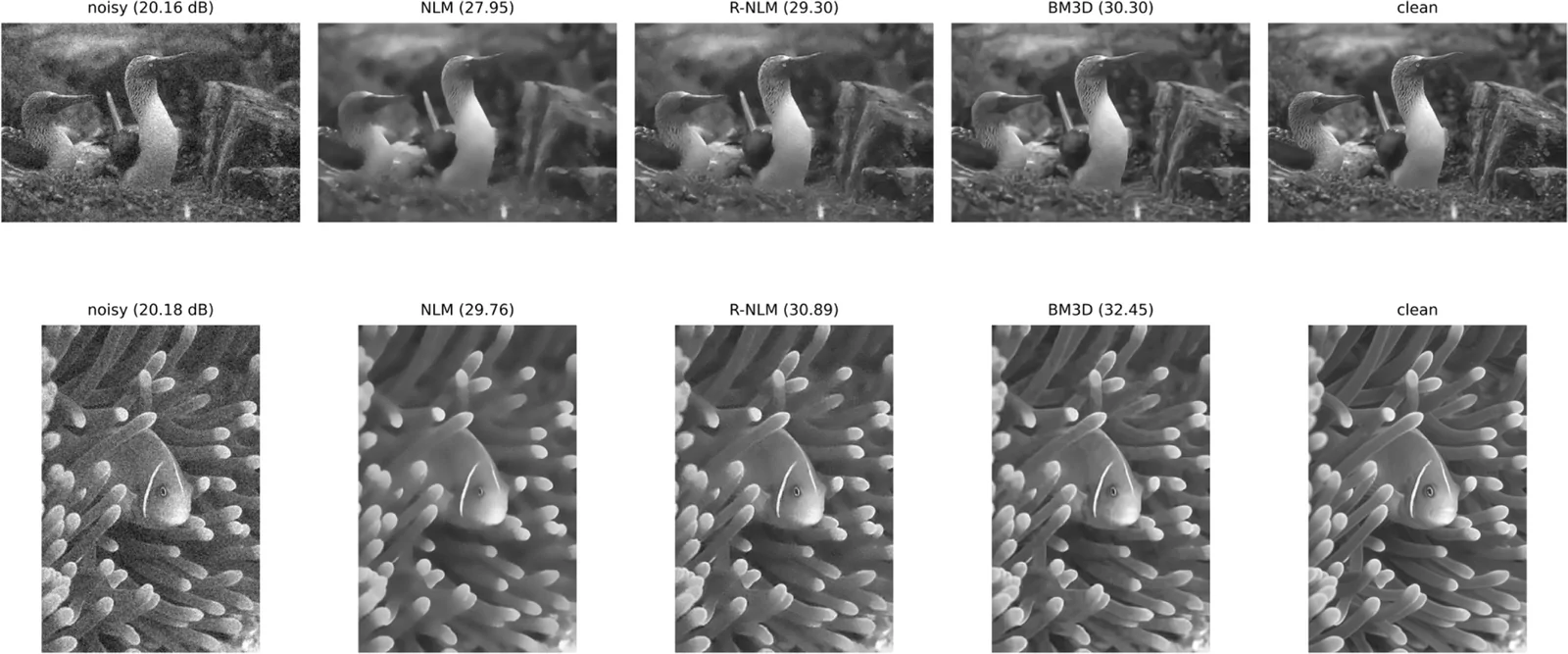
Whole-image comparison, σ = 25, two BSD68 images. From left: noisy, NLM, R-NLM, BM3D, clean. R-NLM removes more noise than NLM while keeping the fine texture that BM3D also preserves.

### Color denoising

The outcome in Table 4 reports the results on color BSD68. The gain over non-local means is larger than in grayscale, 1.86, 2.21, and 2.15 dB at the three noise levels, which is probably due to matching on the smoothed values of all three color channels while one luminance-based weight set averages them; this explanation is an interpretation and was not isolated by a separate ablation. BM3D applied in its color mode is further ahead than in grayscale, by about 2 to 3 dB.

**Table 4:** Color denoising, CBSD68, 20 images, PSNR (dB) / SSIM.

Dataset	Method	σ = 15		σ = 25		σ = 50	
		PSNR	SSIM	PSNR	SSIM	PSNR	SSIM
CBSD68 (20 images)	Noisy	24.61	.582	20.17	.404	14.15	.202
	NLM [8]	29.16	.793	26.73	.709	24.02	.591
	R-NLM	**31.02**	**.888**	**28.94**	**.827**	**26.17**	**.699**
	BM3D-rgb [6,7]	33.87	.933	31.13	.884	27.73	.786
	Δ (R-NLM − NLM)	+1.86	+.094	+2.21	+.119	+2.15	+.108
Kodak24 (24 images)	Noisy	24.61	.518	20.17	.341	14.16	.161
	NLM [8]	29.85	.782	27.42	.707	24.60	.601
	R-NLM	**31.85**	**.873**	**29.77**	**.812**	**26.92**	**.688**
	BM3D-rgb [6,7]	34.43	.919	31.86	.871	28.65	.777
	Δ (R-NLM − NLM)	+2.00	+.091	+2.35	+.105	+2.32	+.087

Color images, PSNR in dB / SSIM. Additive white Gaussian noise on the RGB channels. R-NLM matches on all three channels with a single luminance-based weight; BM3D-rgb uses its opponent-color mode.

### Edge preservation

As seen in Table 5, it lists the edge-preservation index next to PSNR and SSIM on 15 BSD68 images. Relational non-local means lies between non-local means and BM3D on all three measures at every noise level. The gap over non-local means on the edge index widens with noise, from 0.011 at σ = 15 to 0.102 at σ = 50, so the method protects edges most where noise is worse.

**Table 5:** Edge preservation, BSD68 grayscale, 15 images. EPI is the Laplacian-response correlation with the clean image.

σ	Method	PSNR (dB)	SSIM	EPI	ΔEPI vs. NLM
15	NLM [8]	29.43	.807	.706	n/a
15	R-NLM	30.85	.866	**.717**	+.011
15	BM3D [6,7]	31.73	.891	.774	+.068
25	NLM [8]	27.05	.727	.573	n/a
25	R-NLM	28.52	.794	**.606**	+.033
25	BM3D [6,7]	29.22	.827	.659	+.086
50	NLM [8]	24.26	.604	.291	n/a
50	R-NLM	25.38	.650	**.393**	+.102
50	BM3D [6,7]	26.19	.716	.449	+.158

BSD68, 15 images. EPI is the Pearson correlation between the Laplacian responses of the clean and the restored image (1.0 = edges perfectly preserved). R-NLM lies between NLM and BM3D on every measure; its edge advantage over NLM grows with noise.

### Ablation

The report in Table 6 changes one parameter at a time from the fixed setting, on eight BSD68 images, and reports the PSNR gain over non-local means. Two components carry the method. Removing the profiles and matching on the smoothed intensity alone gives a result 0.3 to 2.7 dB below non-local means. Removing the confidence modulation, by setting k = 0, reduces the gain from about 1.3 dB to 0.5 dB at σ = 15 and 25, and to 0.1 dB at σ = 50; reducing the intensity weight to λ = 1, which weakens the anchor, has the same effect. Every other change, to the search radius, the arc taps, the number of scales, and the values of h₀, k, and λ within a moderate range, moves the result by less than 0.2 dB. Outside these ranges, h₀ = 0.45 lowers the gain by about 0.4 to 0.5 dB and K = 16 lowers it by about 0.2 dB. The third sampling radius contributes only at σ = 50. The eight images are those used to select the parameters (Section 1), so the table describes sensitivity around the chosen setting.

**Table 6:** Ablation, PSNR gain over non-local means (dB), BSD68 grayscale, 8 images, at σ = 15 / 25 / 50. A negative value means the output is worse than non-local means.

Configuration	ΔPSNR (σ = 15)	ΔPSNR (σ = 25)	ΔPSNR (σ = 50)	ΔSSIM (σ = 25)
Fixed configuration	+1.29	+1.42	+1.18	+.068
Profiles removed, intensity matching only	-0.31	-0.87	-2.70	-.066
No confidence modulation (k = 0)	+0.48	+0.53	+0.10	+.036
Weak intensity anchor (λ = 1.0)	+0.59	+0.51	+0.09	+.022
Base exponent h₀ = 0.45	+0.80	+0.93	+0.79	+.058
Two sampling radii instead of three	+1.40	+1.39	+1.01	+.064
K = 16 ray directions	+1.11	+1.24	+0.96	+.057
Window R = 5; arc taps = 5; k in [0.4, 1.5]; λ in [1.6, 3.0]; h₀ = 0.75	within ±0.2	within ±0.2	within ±0.2	within ±0.01

BSD68, 8 images. Values are the change relative to NLM (dB for PSNR); a negative value means the output is worse than NLM. One parameter is changed at a time from the fixed configuration. The confidence modulation and the intensity anchor each account for about 1 dB; every other setting stays within 0.2 dB.

### Computational cost

The analysis in Table 7 reports single-image wall-clock time on a laptop processor. The prototype needs 2.2, 3.0, and 3.7 times the time of the compiled scikit-image non-local means at 256 by 256, 512 by 512, and 1024 by 1024 pixels, and BM3D needs 8.5, 5.7, and 4.5 times the time of the prototype. The method therefore has the complexity class of non-local means but is not equally fast in the present implementation. The confidence field accounts for about 40 percent of the time and is written in interpreted array code, so a compiled version would bring the total close to non-local means.

**Table 7:** Wall-clock time per image, seconds. Value in parentheses is the confidence-field share of the R-NLM time.

Resolution	MP	R-NLM, total (s)	R-NLM, C field (s)	NLM (s)	BM3D (s)	R-NLM ÷ NLM	BM3D ÷ R-NLM
256 × 256	0.07	0.40	0.16	0.18	3.4	2.2x	8.5x
512 × 512	0.26	2.1	0.83	0.71	12	3.0x	5.7x
1024 × 1024	1.05	10.4	4.2	2.8	47	3.7x	4.5x

Single image, one laptop CPU (4 threads), numba inner loop. R-NLM has the same asymptotic cost as NLM: O(n·|W|·(1 + KS)) for the weighted average plus O(nKS) for the feature build. The prototype is not fully compiled; the confidence field, about 40 % of the run time, is written in interpreted array code.

### Approaches that did not improve the method

Four extensions were tested and are reported so that they need not be retried. A phase-coherence statistic in place of the cross-scale cosine did not separate texture from noise, with an area under the curve of 0.52 against 0.98 for the cross-scale cosine. A reconstruction that first denoises the field of intensity differences and then solves a Poisson problem for the image converged but plateaued about 3 dB below the confidence-modulated filter. A wrapper that blends a light and a heavy denoise of the input by the confidence field improved Gaussian, total variation, and non-local means outputs by 0.2 to 2.2 dB but slightly reduced the PSNR of BM3D, so it was replaced by the direct relational filter. A second filtering pass with features recomputed on the first output, and larger values of K and S, gave no gain over the single-pass K = 8 form.

## Limitations

The method is 0.7 to 1.4 dB below BM3D on grayscale and about 2 to 3 dB below BM3D in color mode. It has no transform-domain shrinkage stage, and a second filtering pass does not recover the difference. As with other non-local averaging filters, the output can show low-frequency patches in dark, nearly flat regions where the feature distance finds spurious matches. A minimum effective-neighbor count, that is, a wider h(p) wherever the sum of weights is small, is a possible remedy, but it was not implemented or evaluated in this work. The reference implementation is NumPy with a compiled inner loop and runs at 2.2 to 3.7 times the time of a compiled non-local means. The complexity class is the same, and a full compiled implementation is left as an engineering step. Validation uses additive white Gaussian noise. Behavior on real sensor noise, for example the SIDD [13] and DND [14] benchmarks, and a comparison with a trained convolutional denoiser [1,2], are outside the present scope.

## CRediT author statement

**Md. Abid Hasan Rafi**: Conceptualization, Methodology, Software, Validation, Writing - original draft, Writing - review and editing. **Mst. Fatematuj Johora:** Methodology, Data curation, Formal analysis, Validation, Writing – review and editing. **Md. Delowar Hossain:** Investigation, Formal analysis, Writing – review and editing. **Md. Motaharul Islam:** Methodology, Validation, Writing – review and editing. **Md. Murad Kabir Nipun:** Investigation, Visualization, Writing – review and editing. **Tangina Sultana:** Supervision, Project administration, Validation, Writing – review and editing.

## Declaration of competing interest

The authors declare that they have no known competing financial interests or personal relationships that could have appeared to influence the work reported in this paper.

## Data Availability

No data was used for the research described in the article.
